# Synthesis of mesoporous silica from geothermal water

**DOI:** 10.1038/s41598-021-03133-x

**Published:** 2021-12-10

**Authors:** Yujiro Watanabe, Naoki Amitani, Takushi Yokoyama, Akira Ueda, Minoru Kusakabe, Shigeko Unami, Yoshiji Odashima

**Affiliations:** 1 Faculty of Bioscience and Applied Chemistry, Hosei University, 3-7-2 Kajino-cho, Koganei, Tokyo 184-8584 Japan; 2 Faculty of Science, Kyushu University, Motooka 744, Nishi-ku, Fukuoka, 819-0395 Japan; 3 Faculty of Science, University of Toyama, Toyama, 3190, Gofuku, Toyama, 930-8555 Japan

**Keywords:** Design, synthesis and processing, Design, synthesis and processing, Porous materials, Synthesis and processing

## Abstract

Mesoporous silica was successfully synthesized for the first time using geothermal water from the Onuma Geothermal Power Plant, Akita Prefecture, Japan. Cetyltrimethylammonium bromide (CTAB) was used as an organic template for the synthesis. CTAB with a concentration of 2.4 × 10^–4^ mol/L was reacted for 30 min with geothermal water at a temperature of 90 °C, which had a total silicic acid concentration of 475 mg/L (SiO_2_), at pH 7.0, pH 8.2 (raw water) and pH 9.0. By calcination of the resulting precipitate at 550 °C, mesoporous silica with a pore size of about 2.8 nm and a specific surface area of > 800 m^2^/g was formed. The total silicic acid concentration in the solution after formation of the mesoporous precipitates was reduced to < 280 mg/L, indicating efficient recovery of supersaturated silicic acid from geothermal water. The monosilicic acid in geothermal water plays an important role in the formation of mesoporous silica. Production of mesoporous silica by our method will contribute not only to prevention of silica scale formation in the piping systems of geothermal power plants but also to its use as an industrial resource.

## Introduction

In a geothermal power plant, fluids from a deep geothermal reservoir are separated into steam and water. The separated steam is used to rotate a turbine for generation of electricity, whereas the separated water is returned to the underground reservoir through a reduction well. During steam separation, silicic acid is concentrated in the water phase, which becomes supersaturated with respect to the amorphous silica. Polymerization of monosilicic acid followed by reactions between polysilicic and monosilicic acids and between polysilicic acids results in the formation of silica colloids^1–3^. During polymerization, silicic acids precipitate as silica scale inside the pipes of the reinjection wells and the channels in geological formations around the reinjection wells. This results in a reduction of the efficiency of water reinjection, widely known as clogging problems^4–8^. Currently, two methods are in common use to prevent formation of silica scale: (i) pH adjustment and (ii) high-temperature reinjection. In the pH adjustment method, the pH of the geothermal brine is adjusted to around pH 6 to reduce the solubility of amorphous silica and to lower the polymerization rate of silicic acid. In the high-temperature reinjection method, geothermal water is returned to the subsurface at temperatures as high as 150 °C, where the solubility of silica in aqueous solution is high. These methods are unfortunately not fully effective^9–15^. Although some methods to remove silica from supersaturated silicate solution have been proposed^16–18^, they are only partly successful. Thus, it is important to find better and more practical ways for silica removal, followed by its recovery. Additionally, once silica is successfully recovered, its economical use can be envisaged. Mesoporous silica is a useful material in industry and is expected to be applied in a variety of fields, such as catalysts, adsorbents, ion exchangers, optic materials, and solar panels for electric power generation. In general, a cationic surfactant is used to synthesize mesoporous silica, where monosilicic acid is combined with the cationic surfactant. After this process, the combined silicic acids are heated at 80 to 140 °C for dehydration, condensation, and formation of a silica network, which leads to the formation of mesoporous silica precursors. The precursors are acid-washed and calcined to remove the cationic surfactant. The silica thus obtained shows homogeneous mesoporous characteristics. Two pioneering works have reported that the formation of mesoporous silica takes place by means of a liquid–crystal templating mechanism, in which the silicate material forms inorganic walls between ordered surface micelles^19,20^. Subsequent publications have defined the mechanism as macro-molecular templating, initiated by silicate anions and involving cooperative self-assembly upon mutual attraction between silicate and surfactant ions^21–23^. MCM-41, MCM-48, and MCM-50 from the M41S family of mesoporous silica are synthesized by adding cetyltrimethylammonium bromide (CTAB) as a cationic surfactant^24^. The mixture is matured in an alkaline solution (ca. pH 11) and processed under hydrothermal conditions at 100 °C^25^. The mesoporous silica synthesized in this way has a high specific surface area of ca. 1000 m^2^/g.

We investigated the possibility of direct synthesis of mesoporous silica from supersaturated silicic acid dissolved in high-temperature geothermal water. The use of geothermal water has advantages in that the high water temperature (ca. 100 °C) can supply sufficient thermal energy for the synthesis of mesoporous silica, and that monosilicic acid is dissolved at high concentration. To date, there have been many articles on the synthesis of mesoporous silica by adding CTAB to silica scale formed in pipes in geothermal plants^26–29^. There have been no reports on the direct synthesis of mesoporous silica from geothermal fluids. Kitsuki et al.^30^ described the selective removal of polysilicic acid from geothermal water at the Hatchobaru Geothermal Power Plant, Oita, Japan, by using CTAB, and indicated that dissolved silica in geothermal fluids can be effectively recovered. However, their report did not mention the formation of mesoporous silica.

In the present study, we examined conditions under which mesoporous silica can be directly synthesized from geothermal fluids at the Onuma Geothermal Power Plant. Since silicic acid exists as monosilicic acid at the time of sample collection, the formation processes for mesoporous silica with the cationic surfactant can be determined. It is conceivable that a silica network could be formed through dehydration and condensation-polymerization in the presence of CTAB, a process similar to the formation process for polysilicic acid in a high-temperature solution^2,31^. We investigated the change in silicic acid concentration over time when CTAB was added to geothermal water. We also examined the properties of precipitates that were recovered from the geothermal water at different pH conditions.

## Results and discussion

### Variation of monosilicic acid concentrations after sampling of geothermal water

**Figure 1:**
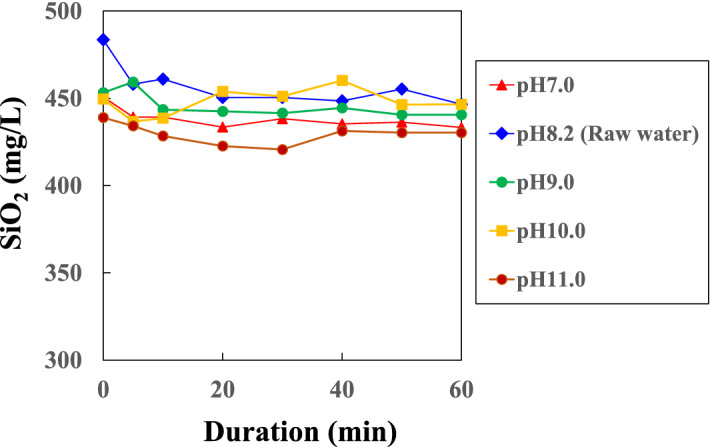
Change over time of monosilicic acid concentration in tested solutions.

Figure 1 shows the variation with time of the concentrations of monosilicic acids in the Onuma geothermal water at pH 7 to 11 after sampling. The concentrations of each were similar, and little change with time was observed. More than 420 mg/L of monosilicic acid was present, indicating that most of the silicic acid was present as monosilicic acid. This suggests that polymerization of silicic acids was slow in the sampled geothermal water. The solubility of silicic acid in water at 90 °C is 380 mg/L^32^ and the Onuma water is supersaturated at approximately 95 mg/L. At such a low degree of silica supersaturation, the polymerization rate for monosilicic acid is low. Since monosilicic acid needs to be combined with CTAB at the initial stage of mesoporous silica formation, the geothermal fluid used in this study is suitable for the formation of mesoporous silica.

### Characteristics of silica precipitates and their calcined products

**Figure 2:**
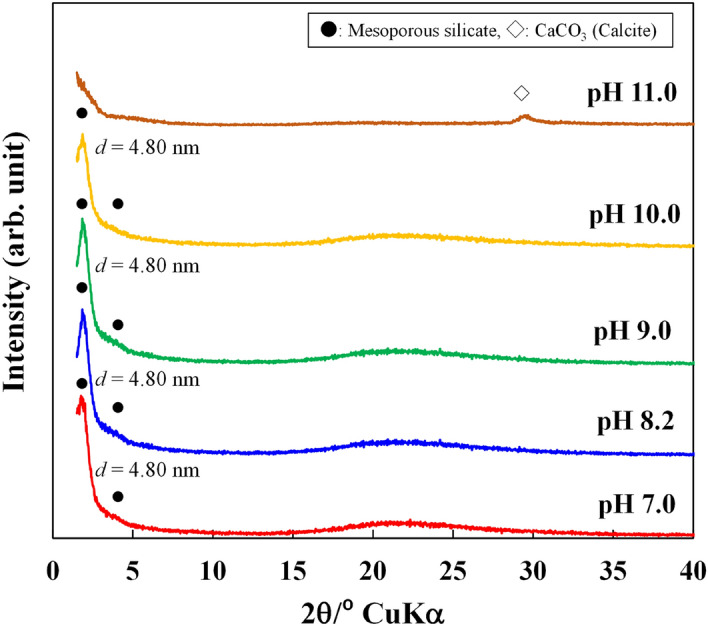
XRD patterns for precipitates.

Figure 2 shows X-ray diffraction (XRD) patterns for precipitates obtained 30 min after addition of CTAB. The precipitates from the solutions with pH 7–10 produced a diffraction peak at a 2*θ* value of 1.84°, indicating a *d* value of 4.80 nm, which is typical for mesoporous silica. The XRD intensity for the samples obtained at pH 7.0, 8.2 and 9.0 was strong. At pH 11.0, however, no diffraction peak around 1.84° was observed, but a weak peak due to CaCO_3_ (calcite) appeared at 29°. CaCO_3_ was formed by the reaction between Ca^2+^ and CO_3_^2−^ ions that were present at low concentrations under alkaline conditions. The precipitates obtained after a 30 min reaction with CTAB at 30 and 60 minutes after water sampling showed essentially the same XRD patterns as those obtained after a 30 min reaction with CTAB, which was added immediately after water sampling.

**Figure 3:**
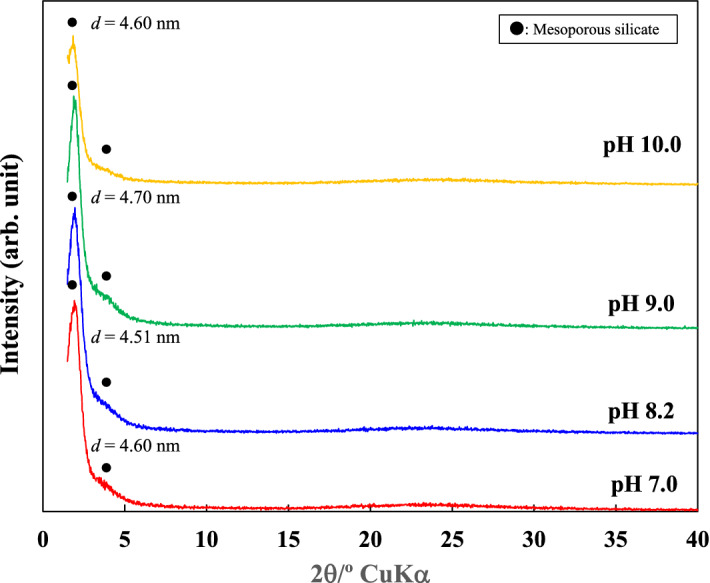
XRD patterns for calcined precipitates.

Figure 3 shows the XRD patterns for the calcined products that were obtained after the reaction of geothermal water with CTAB for 30 min. Calcination removed cetyltrimethylammonium ion (CTA^+^) templates and induced the formation of fine pores, which was supported by the observation that the sharp XRD peak at around 1.9°, indicating a *d* value of around 4.65 nm, remained and a peak at 4.2° was observed, although it was quite broad. This suggests that fine pores were produced. M41S, which is a typical mesoporous silica with one-dimension channels, has two XRD peaks^24^. In the present study, the mesoporous silica obtained also produced two XRD peaks and a peak pattern similar to that for M41S. These results suggested that M41S was directly produced from geothermal water.

In general, the M41S family is produced through hydrothermal synthesis of a silica-supersaturated (gel-suspended) solution by adding CTAB at more than its critical micelle concentration^25,33^. We used a CTAB concentration lower than the critical micelle concentration since the geothermal water was dilute with respect to silicic acid. In our case, M41S was formed by adding CTAB at lower concentration. The mechanism suggested macro-molecular templating, initiated by silicate anions and involving cooperative self-assembly upon mutual attraction between silicate and surfactant ions^21–23^. However, the intermediate structures of interacting entities are unknown. Huo et al.^23^ reported that mesoporous silica could be produced from a solution with a low surfactant concentration. This suggests that the interaction between organic and inorganic units is effective in the formation of the mesoporous structure, i.e., the CTA^+^ positive charge attracts monosilicic acid molecules. These organic and inorganic units are combined, self-assembled and polymerized to become a precursor with a mesoporous structure.

**Figure 4:**
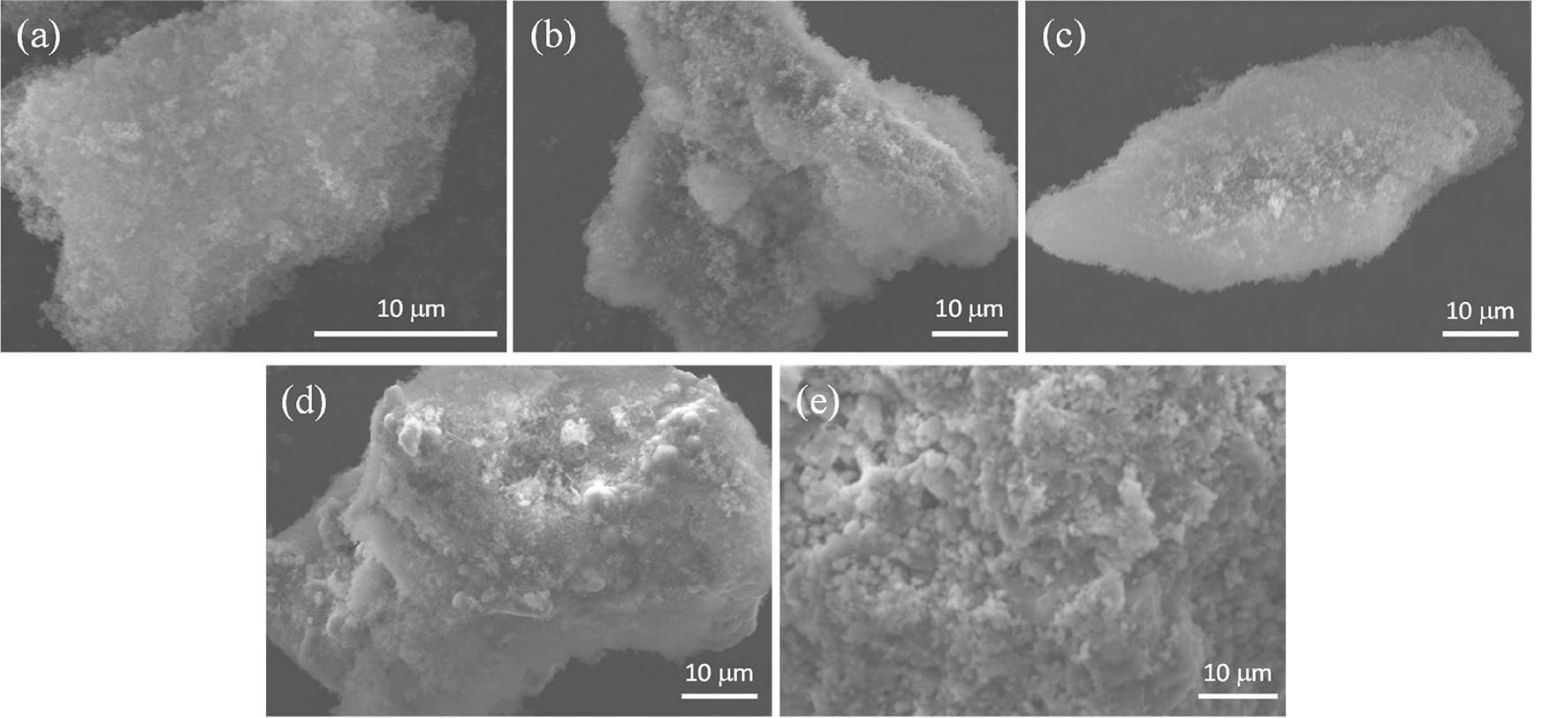
SEM images of precipitates obtained at (**a**) pH 7.0, (**b**) pH 8.2, (**c**) pH 9.0, (**d**) pH 10.0 and (**e**) pH 11.0.

**Figure 5:**
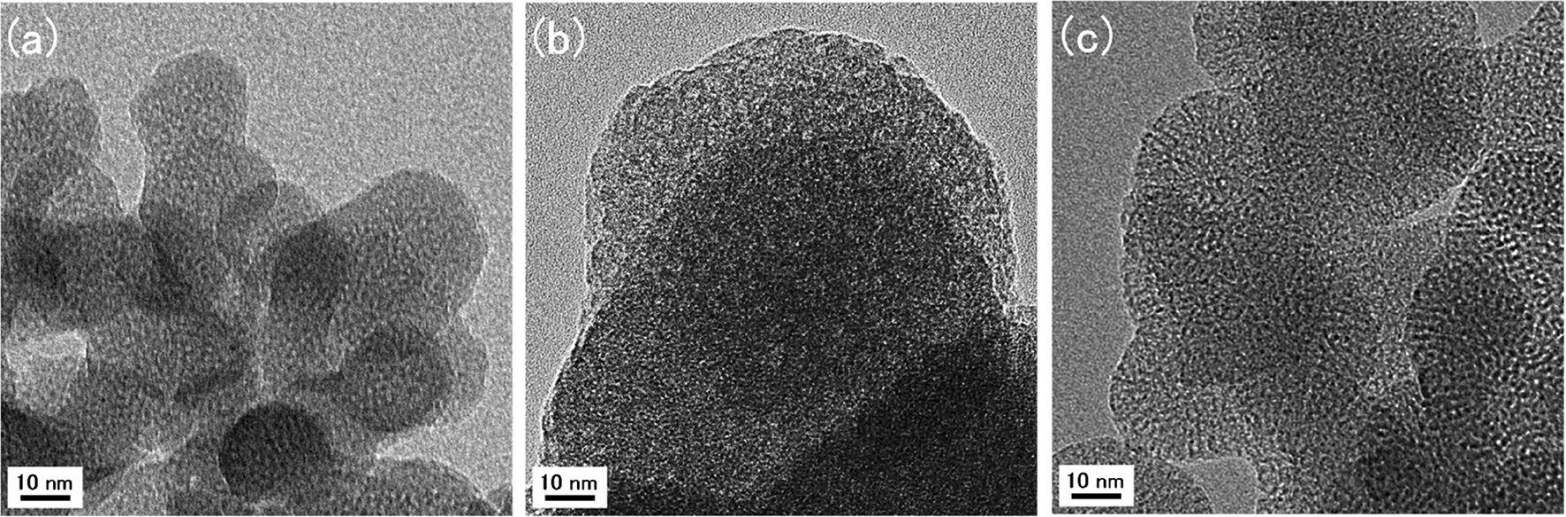
TEM images of calcined precipitates obtained at (**a**) pH 7.0, (**b**) pH 8.2 and (**c**) pH 9.0.

**Figure 6:**
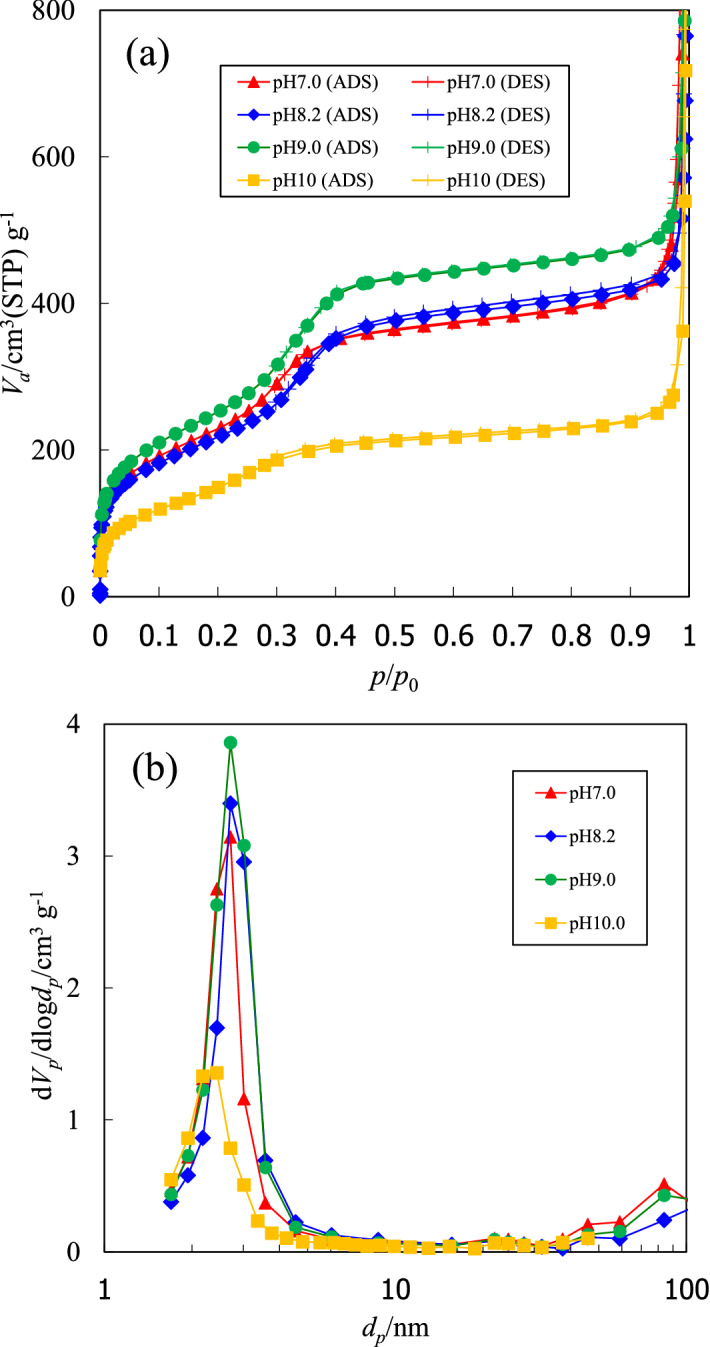
(**a**) Nitrogen adsorption/desorption isotherms for calcined precipitates, and (**b**) BJH plots for pore size distributions.

Scanning electron microscopy (SEM) images of precipitates from the experiments at pH 7.0, 8.2, 9.0, and 10.0 showed aggregates of amorphous M41S microcrystals, whereas the precipitate from the experiments at pH 11.0 showed rhombohedral-like calcite (CaCO_3_) crystals (Fig. 4). Transmission electron microscopy (TEM) images of calcined precipitates from the experiments at pH 7.0, 8.2 and 9.0 showed mesoporous structures with pore sizes of 2–3 nm (Fig. 5). The distance between each pore (wall thickness) was 1–2 nm. The M41S does not have a hexagonal structure, which is a typical characteristic of MCM-41. The structure is unknown^24^. The specific surface areas, mean pore diameters and pore volumes for the calcined precipitates formed at pH 7–10 are shown in Table 1. These values were obtained from isotherms of nitrogen adsorption and desorption experiments. Barrett–Joyner–Halenda (BJH) plots for pore sizes of less than 50 nm were calculated from the isotherms (Fig. 6). The isotherms were typical of mesoporous materials. The specific surface areas of the precipitates obtained from the pH 7.0, 8.2, and 9.0 experiments were greater than 800 m^2^/g. Table 2 shows the results of an energy-dispersive X-ray spectroscopy (EDX) analysis (excluding carbon) of the precipitates obtained under different pH conditions. The precipitates from the pH 7–10 runs contained Si and O as main components, suggesting that the precipitates were composed of SiO_2_. Although a minute amount of Al substituted in Si sites was detected, other ions were not present. This suggested the formation of mesoporous silica of high purity. The precipitates from a pH 11.0 run contained an appreciable amount of Ca ions, implying CaCO_3_ formation, consistent with the XRD and SEM analysis results. Mesoporous silica is generally synthesized under highly alkaline conditions where reactions between the monosilicic acid and CTAB surfactant are promoted^25^. It has been suggested that this may inhibit the reaction between CTA^+^ and monosilicic acid. The arsenic concentration in the precipitates was below the detection limit (0.01%). This is favorable for production of mesoporous silica on an industrial basis.

**Table 1:** Surface areas, pore diameters and pore volumes for mesoporous silica obtained in different pH solutions.

pH	Surface area (m^2^/g)	Pore diameter (nm)	Pore volume (cm^3^/g)
7.0	834	2.8	0.6
8.2	814	2.8	0.6
9.0	919	2.8	0.7
10.0	551	2.6	0.4

**Table 2:** Chemical composition (wt%) of mesoporous silica obtained in different pH solutions.

pH	SiO_2_	Al_2_O_3_	Ca	Na	K	Cl
7.0	46.2	2.23	0.00	0.02	0.00	0.23
8.2	44.5	2.25	0.08	0.13	0.15	0.27
9.0	48.9	2.29	0.02	0.00	0.03	0.13
10.0	45.8	3.78	0.04	0.01	0.02	0.07
11.0	35.5	1.11	16.61	0.65	0.05	0.30

From the foregoing discussions, we can conclude that the synthesis of mesoporous silica is best achieved under neutral to weakly alkaline conditions, e.g., at pH 7–9, where silicic acid exists mainly as monosilicic acid. In addition, no impurities such as calcite and arsenic acid were incorporated under pH conditions of 7 to 9.

### Formation process for mesoporous silica in geothermal water

**Figure 7:**
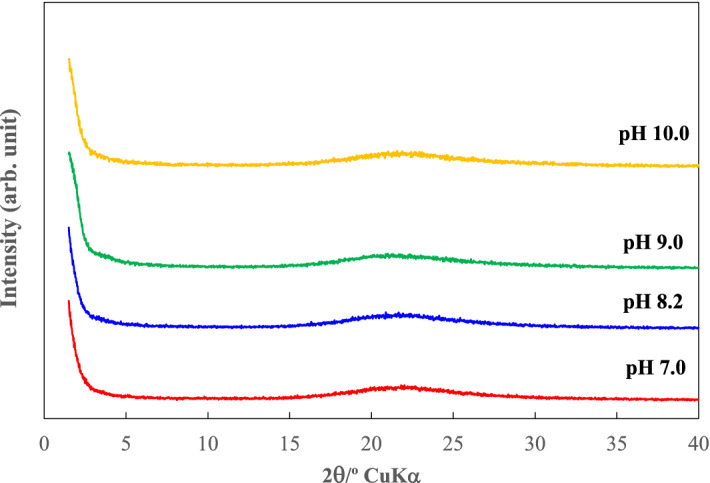
XRD patterns for precipitates prepared in the laboratory.

Cetyltrimethylammonium ions (CTA^+^) combine preferentially with dissociated silanol groups (–Si–O–) on the surface of polysilicic acid but not with monosilicic acid^30^. In our experiments, however, the CTAB concentration was high and monosilicate ions were attracted to the positive charges on the surface of the CTA^+^. In the laboratory, the same experiment was conducted using geothermal water (pH 7–10) that was more than 4 weeks old. The concentration of monosilicic acid in the geothermal water was less than 150 mg/L, and most of the silicic acid was present as polysilicic acid. CTAB was added to the geothermal water at a concentration of 2.4 × 10^–4^ mol/L and kept in a water bath at 90 °C for 30 min. The XRD patterns of the precipitates showed no diffraction peaks attributed to mesoporous silica (Fig. 7). It is considered that the formation of mesoporous structure was inhibited by the large amount of polysilicic acid in the geothermal water. The polysilicic acid may have condensed to form amorphous silica in this condition. Thus, it is considered that the combination of monosilicic acid with CTA^+^ plays an important role in the formation of a mesoporous structure. We have clearly shown that mesoporous silica can be synthesized in the presence of a small amount of CTAB in geothermal water containing a large proportion of monosilicic acid at pH 7–9.

### Effect of CTAB addition on the change in silicic acid concentration

**Figure 8:**
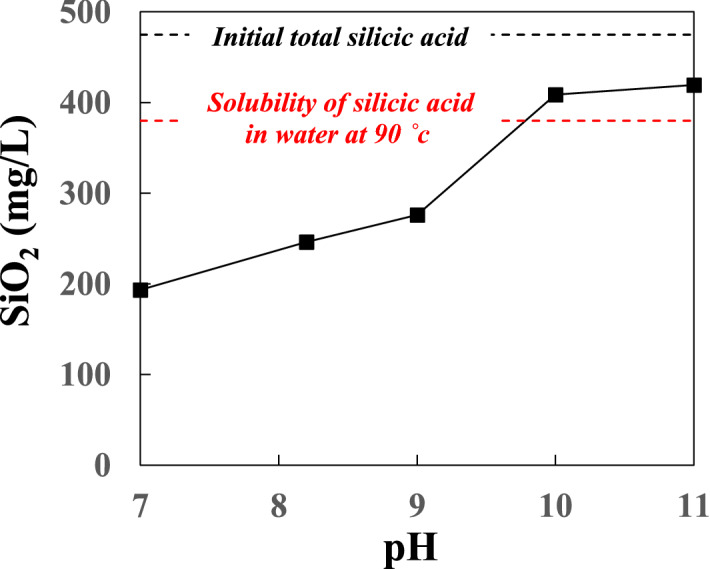
Total silicic acid concentration 30 min after CTAB addition.

The change with time of the total silicic acid concentration in geothermal waters 30 min after the addition of CTAB at pH 7–11 is illustrated in Fig. 8. The silicic acid concentration was most reduced at pH 7.0, and it increased with increasing pH. At pH 10.0 and 11.0, the reduction of the silicic acid concentration was small compared to the initial total silicic acid concentration of the raw geothermal water (475 mg/L at pH 8.2), indicating that a large proportion of silicic acid remained unreacted. At lower pH, mesoporous silica having a high specific surface area was synthesized. The total silicic acid was reduced by 229 mg/L compared to the initial total value, indicating that the recovery rate for the total silicic acid was 48.2%. We were able to recover more silica than the saturated concentration of 380 mg/L^32^, the solubility of amorphous silica at 90 °C. The excess silica recovered from the tested thermal water amounted to 95 mg/L. Our results show that high-quality mesoporous silica can be synthesized from geothermal waters, and that our technique can be applied to the reduction of silica scale at geothermal power plants. The reaction with CTAB can also remove Al from geothermal waters, thus contributing to the simultaneous removal of Al and silica, which prevents scale formation in piping systems^34,35^.

## Conclusions

We succeeded in the first direct synthesis of mesoporous silica using thermal water from the Onuma Geothermal Power Plant, Akita, Japan. Mesoporous silica was synthesized by adding CTAB at a concentration of 2.4 × 10^–4^ mol/L to the water at 90 °C, waiting for 30 min for completion of the reaction, filtering and drying the precipitates, and then calcinating at 550 °C. The mesoporous silica obtained from pH 7.0, 8.2, and 9.0 geothermal water had a high specific surface area of > 800 m^2^/g. It is expected that excess dissolved monosilicic acid can be removed and recovered as mesoporous silica. Thus, scale formation can be prevented. Our new technique demonstrates that mesoporous silica can be directly produced from geothermal water, leading to prevention of silica scale and to the production of economically valuable materials.

## Methods

### Sampling and chemistry of thermal water

The experiments were performed between October 29th and November 1st, 2020, at the Onuma Geothermal Power Plant operated by Mitsubishi Materials Corporation, Akita Prefecture, Japan. The two-phase thermal water from the production well O-12R was separated into steam and water using a separator. The chemical composition of the water phase is shown in Table 3. The water was relatively dilute with pH 8.2, [Na^+^] = 406 mg/L, [K^+^] = 41 mg/L, and [Ca^2+^] = 15.5 mg/L. The total SiO_2_ concentration was 475 mg/L. The temperature of the water was 90 °C at the time of sample collection. Since the solubility of amorphous silica at 90 °C is 380 mg/L^32^, SiO_2_ existed in excess by 95 mg/L. The Al^3+^ concentration in the sample solution was 0.8 mg/L.

**Table 3:** Chemical composition of geothermal water used to synthesize mesoporous silica at well O-12R, Onuma geothermal power plant.

Dissolved species	Concentration (mg/L)
Na^+^	406
K^+^	41.2
Ca^2+^	15.5
Mg^2+^	< 0.01
Fe^2+^	< 0.01
Al^3+^	0.8
Cl^−^	431
SO_4_^2−^	225
HCO_3_^−^	86
SiO_2_	475
pH	8.2
EC (mS/m)	201

Data supplied by Mitsubishi Material Co. Ltd. Date of sampling: 2020/7/21.

### Synthesis of mesoporous silica from geothermal water

Twenty liters of sample water (pH 8.2) were collected in a polyethylene container. CTAB was added to the geothermal water to a concentration of 2.4 × 10^–4^ mol/L. The water was kept in a water bath at 90 °C for 30 min. The CTAB concentration was higher than 1.0 × 10^–4^ mol/L, which was sufficient to react with Si–O units that existed on the surface of polysilicic acid^26^. Similar experiments were performed at pH 7.0, 9.0, 10.0, and 11.0, adjusted using either H_2_SO_4_ or NaOH solutions. Thirty minutes later, the precipitates were filtered through filter paper and dried at room temperature using a vacuum drying oven. In the laboratory, the same experiment was carried out using geothermal water (pH 7–10) that was more than 4 weeks old. CTAB was added to this geothermal water at a concentration of 2.4 × 10^−4^ mol/L. The water was kept in a water bath at 90 °C for 30 min. For raw geothermal water (pH 8.2), CTAB was added 30 or 60 min later. The reaction time was 30 min.

### Analytical procedures

#### Analysis of mono- and total silicic acid concentrations

850 mL of untreated geothermal water was placed in a 1 L Teflon beaker to which 3 mL sulfuric acid or NaOH solution had been added to adjust the pH of the solution to 7.0, 9.0, 10.0 or 11.0 (± 0.2). The solution was kept at 90 °C for 30 min. An aliquot of the solution was sampled at intervals of 5 to 10 min and filtered through a 0.22 μm membrane filter. The monosilicic acid concentration was measured spectrophotometrically (UV-1280; Shimadzu) at 385 nm based on the formation of a yellow molybdosilicic acid complex. The total silicic acid concentration was obtained after making the solution alkaline by adding NaHCO_3_ powder and heating. This process decomposed polysilicic acid to monosilicic acid for which the concentration was determined spectrophotometrically. The analytical precision was ± 5%. The total silicic acid concentration after 30 min reaction with CTAB was determined similarly using an ICP emission spectrometer (ICPE9800; Shimadzu).

#### Characterization of silica precipitates

The dried silica precipitates were checked for their crystallinity using powder XRD (Ultima IV; Rigaku) with monochromatized CuKα radiation operated at 40 kV and 30 mA, for their appearance using TEM (JEM-2000FX; JEOL) operated at 200 kV and SEM (JSM-6700; JEOL) operated at 15 kV, and for their chemical compositions using EDX (EMAX; Horiba). CTAB was removed by gradual heating of the precipitates in an electric furnace to 550 °C (temperature was raised at a rate of 10 °C/min) for 1 h under nitrogen atmosphere. The precipitates were calcined at 550 °C in air for an additional 1 h. The sample thus obtained was analyzed with XRD. The specific surface areas, pore diameters and pore volume were measured using a nitrogen adsorption and desorption apparatus (BELSORP MINI X; MicrotracBEL) after removal of physically adsorbed water at 200 °C for 6 h in vacuum. These values were calculated from the nitrogen adsorption and desorption isotherms using the Brunauer–Emmett–Teller (BET) method and the BJH method^36^.
